# Efficacy of 0.01% atropine for myopia control in a randomized, placebo-controlled trial depends on baseline electroretinal response

**DOI:** 10.1038/s41598-022-15686-6

**Published:** 2022-07-08

**Authors:** Henry H. L. Chan, Kai Yip Choi, Alex L. K. Ng, Bonnie N. K. Choy, Jonathan Cheuk Hung Chan, Sonia S. H. Chan, Serena Z. C. Li, Wing Yan Yu

**Affiliations:** 1 Laboratory of Experimental Optometry (Neuroscience), School of Optometry, The Hong Kong Polytechnic University, 11 Yuk Choi Road, Hung Hom, Kowloon, Hong Kong; 2 Centre for Myopia Research, School of Optometry, The Hong Kong Polytechnic University, Kowloon, Hong Kong; 3 Research Centre for SHARP Vision (RCSV), The Hong Kong Polytechnic University, Kowloon, Hong Kong; 4 Department of Ophthalmology, LKS Faculty of Medicine, The University of Hong Kong, Pokfulam, Hong Kong; 5 Centre for Eye and Vision Research Limited, 17W Hong Kong Science Park, Shatin, Hong Kong; 6 University Research Facilities in Behavioral and Systems Neuroscience (UBSN), The Hong Kong Polytechnic University, Kowloon, Hong Kong

**Keywords:** Public health, Predictive markers, Refractive errors

## Abstract

This study aimed to evaluate the efficacy of 18-month 0.01% atropine in 61 myopic children (aged 7–10) and the relationship with central retinal response (by multifocal electroretinogram [mfERG]) in a double-masked randomized placebo-controlled clinical trial. Global-flash mfERG was measured at baseline, while cycloplegic spherical equivalent refraction (SER) and axial length (AL) were measured at baseline and at 6-month intervals. Annualized change in SER and AL were compared between atropine and control groups, and the relationships with baseline mfERG were evaluated. Changes in SER (−0.70 ± 0.39D vs. −0.66 ± 0.41D, p = 0.63) and AL (0.32 ± 0.16 mm vs. 0.30 ± 0.22 mm, p = 0.52) were similar in atropine and control groups. Interestingly, in the placebo group, mfERG amplitude was negatively correlated with axial elongation (R_p_ = −0.44, p = 0.03) as in our previous study. However, in the atropine group, an opposite trend was observed that axial elongation was positively correlated with mfERG amplitude (R_a_ = 0.37, p = 0.04). Annualized myopia progression demonstrated similar opposite effect between atropine and placebo groups but did not reach statistical significance. An ERG screening protocol may be warranted to identify suitable candidates to reduce the likelihood of an unfavorable treatment response by 0.01% atropine.

## Introduction

Myopia prevalence has been soaring in recent decades, reaching pandemic levels^1^ globally^2^. This has significant public health implications because high myopia is associated with increased risk of sight-threatening complications^3^. To prevent myopia progression, various non-pharmacological interventions are available including multifocal contact lenses^4^, simultaneous defocus lens^5^, orthokeratology^6^, and defocus-incorporated spectacle lenses^7^. In addition, based on the reported efficacy for myopia control, low-concentration atropine eyedrops are currently accepted and commonly used as a pharmacological intervention to limit myopia progression in children^8–10^. Relative to formulations with higher atropine concentrations, 0.01% atropine has been associated with fewer side effects in children (including mydriasis and reduced accommodation), less rebound-myopia after treatment cessation, but comparatively weaker efficacy in the first year of application^11^.

Historically, high-concentration atropine (1.0%) was found to effectively control myopia progression by a landmark study—Atropine for the Treatment of Myopia (ATOM)^12^. However, due to significant adverse effects, including cycloplegia, mydriasis, and rebound on cessation, the long-term safety of 1.0% atropine has been a concern. Subsequent studies focused on optimizing the dose of atropine to achieve a more favorable risk/benefit profile^8–10^. The ATOM2 study tested various concentrations of atropine, and atropine 0.01% was included as pseudo-placebo control under the assumption that it has minimal efficacy. While atropine efficacy in ATOM2 dose-dependently increased, myopia progression in the atropine 0.01% group was lower than in the placebo group of the ATOM1 study, suggesting that it was efficacious in reducing myopia progression. However, in the Low-Concentration Atropine for Myopia Progression (LAMP) Study, 0.01% atropine did not show significant reduction in axial elongation^9, 13^. Instead, recent studies have shown that higher atropine concentrations, such as 0.02% and 0.05%, have better efficacy and been strongly advocated^9, 10^.

Electroretinography (ERG) is an objective method to quantitatively evaluate the retinal function by stimulating retinal neurons. Multifocal ERG (mfERG) allows localized measurements to evaluate responses in different regions of the retina, but in large part measures the response of the outer retinal layers containing photoreceptors and bipolar cells^14, 15^. The global-flash mfERG (MOFO mfERG), which incorporates a periodic bright frame within conventional mfERG stimulation, selectively enhances the detection of the inner retinal response^16, 17^. Previous studies revealed that the foveal MOFO mfERG at baseline predicted the subsequent change in refractive error in emmetropic children with normal visual acuity^18, 19^. Specifically, children with a weak central inner retinal MOFO mfERG response had more severe myopia progression subsequently, showing that subclinical impairment of retinal function, as measured by mfERG, may be a predictor for myopia development.

Because of its favorable safety profile, topical 0.01% atropine has been a popular choice among the available interventions for school-aged children with rapid myopia progression. While the targeting tissue and mechanism for myopia control were elusive, retina was one of the suggested origins of action. However, the interaction between atropine and retinal function on myopia development remains unclear, given that our previous findings suggested weakened retinal function precedes myopia development^18, 19^. The current double-blind randomized clinical trial was designed to investigate the relationship between the baseline MOFO mfERG and the subsequent change in refractive error following treatment with 0.01% atropine or placebo.

## Materials and methods

### Study design

The current study adopted a double-blind, randomized, parallel group, placebo-controlled design, to investigate the MOFO mfERG as a predictor of response to 0.01% atropine for myopia control, as well as the efficacy of 0.01% topical atropine on myopia progression in overall in school-aged children (ClinicalTrials.gov Identifier: NCT03374306, first registered on 15/12/2017). The trial registration information is available at https://clinicaltrials.gov/ct2/show/NCT03374306. The primary endpoint of the study was annualized change in spherical equivalent refraction (SER), and the secondary endpoint was annualized change in axial length (AL). The relationships between baseline retinal electrophysiological response and SER and AL were also analyzed such that the interaction of baseline MOFO mfERG with atropine treatment was evaluated. All the procedures followed the Tenets of Declaration of Helsinki and were approved by the Human Subjects Sub-committee of The Hong Kong Polytechnic University. This study followed the guidelines of Consolidated Standards of Reporting Trials (CONSORT).

### Study population

The study was advertised, and recruitment invitations were sent to parent groups, describing the details of the clinical trial. Prior to any study-specific procedure, written informed consent and verbal assent were obtained from parents and participants, respectively. The participants were 7 to 10 years of age (inclusive), having a SER between −0.50 D and −5.00 D inclusive and an astigmatic error of less than 1.00 DC. The participants were required to have normal best-corrected visual acuity (LogMAR 0.00 or better) and normal color vision. Any strabismus, amblyopia, ocular, or systemic disease, and a history of epilepsy were exclusionary.

### Randomization, masking, and intervention

After confirmation of the eligibility, all participants were randomly assigned in a 1:1 ratio to either 0.01% atropine or placebo treatment. The randomization process was conducted using a computer-generated random sequence by one investigator (HHLC) while all other investigators and participants were masked. The atropine eyedrops, manufactured by AIM (Aseptic Innovative Medicine Co. Ltd, Taiwan), contained 0.01% atropine, while the placebo eyedrop contained 0.9% sodium chloride. All the packages were the same for both atropine and placebo eyedrops to keep the participants and other investigators masked. Participants were prescribed a 3-month dosing of single-dose eyedrops at a quarterly interval and were asked to instill the assigned eyedrops once every day in both eyes.

### Sample size and power

In a previous study with low-concentration atropine, the annual myopia progression rate (mean ± SD) under 0.01% atropine and placebo control were −0.49 ± 0.63 D and −1.20 ± 0.69 D, respectively, equivalent to an effect size of 1.07^20^. Therefore, 24 participants per treatment group were predicted to provide 95% power (5% Type I error, two-sided test). Assuming a dropout rate of approximately 20% throughout the study period (based on prior experience), the minimal enrollment goal was 60 participants.

### Study procedures

Baseline and follow-up eye examinations were performed at the Optometry Research Clinic of The Hong Kong Polytechnic University, including refraction, axial length, and mfERG assessments (KYC, WYY, and SZCL). Additional visits to the Eye Clinic in Grantham Hospital, Hong Kong were arranged for ophthalmological consultations (ALN, BLC, or JCHC) and collection of the pre-assigned eyedrops (0.01% atropine or placebo). Participants and their parents were instructed to instill 1 drop of the assigned eyedrops to both eyes once every 24 h. The participants were asked to return to the hospital eye clinic at 3-month intervals and to the university optometry clinic at 6-month intervals throughout the 18-month study period. The recruitment started from Feb 2018 and the last follow-up visit ended in May 2020. Due to COVID-19-related lockdowns, a notable proportion of follow-up assessments was performed outside the pre-specified visit window, which was accounted for by calculating annualized change in SER and AL.

### Outcome measures

Refraction was measured for at least 5 times using an open-field auto-refractor (NVision K5001, Shin-Nippon, Japan) 30 min after instillation of cycloplegic agent (2 drops of 1% cyclopentolate, 5 min apart). AL was measured for at least 5 times using an IOLMaster (Carl Zeiss, Dublin, CA). Owing to the strong correlation between right and left eyes (SER r = 0.82, p < 0.001; AL r = 0.94, p < 0.001), only right eye was reported to simplify the statistics.

**Figure 1:**
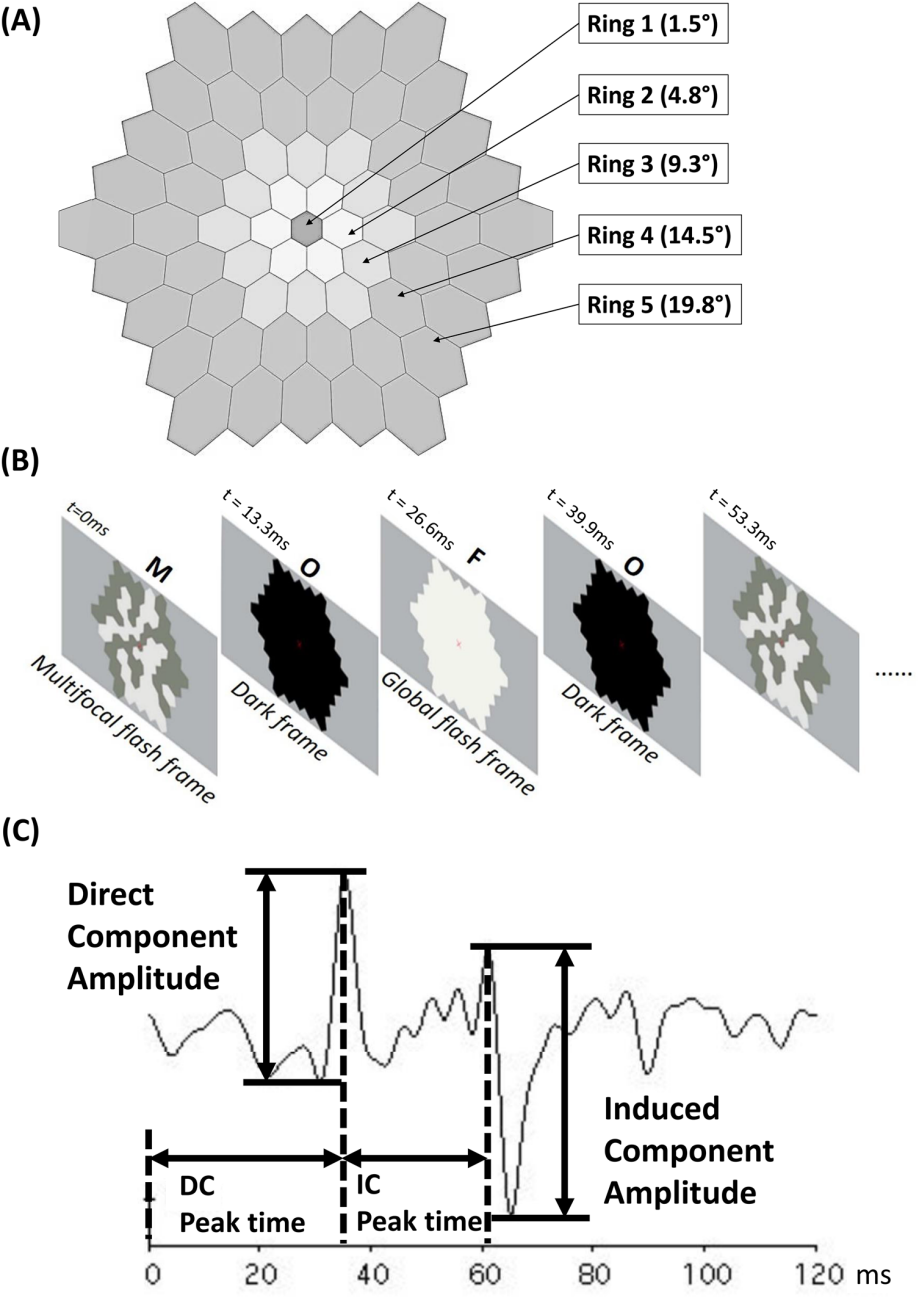
Schematic diagram showing the pattern of MOFO mfERG stimulation. (**A**) Stimulation pattern coverage and regions. (**B**) Video frame sequence. (**C**) A typical MOFO mfERG response with direct and induced components.

The retinal activity was measured by MOFO mfERG (VERIS Science 6.0.6d19, Electro-Diagnostic Imaging, Milpitas, CA) with a minimum pupil diameter of 7 mm. A Dawson-Trick-Litzkow electrode was used as active electrode (positioned between the cornea and palpebral conjunctiva), and gold-cup surface electrodes were used as the reference and ground electrodes (positioned at the outer canthus of the tested eye and on the forehead, respectively). The participants were exposed to the stimulus pattern displayed on a 22-inch monitor with 75 Hz frame rate (VG2239M-LED, ViewSonic, Brea, CA) at 40-cm working distance, consisting of 61 hexagons, and subtended 37° horizontally and 33° vertically (Fig. 1A), with a 40-cm-adjusted sphero-cylindrical correction based on the cycloplegic refraction. The stimulation cycle started with a multifocal frame (M), followed by a dark frame (O), a global flash frame (F), and finally a second dark frame, i.e., MOFO, repeated for 2^12^–1 sequences (Fig. 1B). The luminance for the bright and dark hexagons were 140 and 48 cd/m^2^, respectively, to achieve a 49% contrast with background mean luminance of 94 cd/m^2^. The recording bandpass was set from 10–200 Hz with 100 k times signal gain. The responses were pooled into 5 concentric regions: Rings 1 to 5 (Fig. 1A), in which the direct and induced components (DC and IC) were determined, and the amplitudes and peak times were measured (Fig. 1C).

### Statistical analysis

Due to COVID-19-dependent closure of the university campus, the eye examinations were not always performed within the study-defined time window and were in-part delayed. To minimize the effect of delayed visits, change from baseline in SER and AL were normalized for time and expressed as annualized change for each participant. All participants included in the analysis had completed the baseline eye examination and at least 2 visits of follow-up eye examinations during the study period.

The data distribution was approximately normal (Shapiro–Wilk test—SER: p = 0.16; AL: p = 0.27). A univariate general linear model (GLM) was used to compare annualized change in SER and AL between the treatment groups (atropine vs. placebo), controlling for the baseline SER and age as covariates. Since a weakened central inner retinal response was reported as a risk factor for myopia development^18^, the relationship between Ring 1 IC and annualized change in SER and AL were evaluated using GLM, and compared between the atropine and the placebo groups using moderator analysis. The correlation coefficients (R_p_ vs. R_a_) were also compared between treatment types using Fisher’s R-to-Z test in the Ring 1 IC, as well as other regions and MOFO mfERG parameters. All statistical procedures were performed with SPSS22.0 (IBM, Armonk, NY). Hochberg’s adjustment was applied for multiple comparisons^21^, with significance level set at p ≤ 0.05. A receiver operating characteristics analysis was also performed to evaluate the predictive value of baseline Ring 1 IC on fast progressor (annualized progression ≥ 1.0 D) in each of the atropine and control group.

## Results

**Figure 2:**
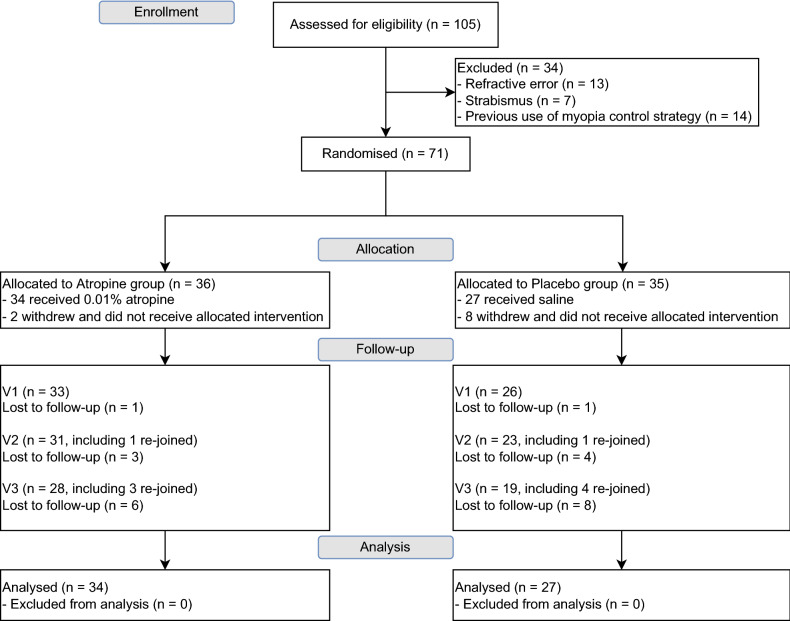
CONSORT flow diagram.

Of 105 screened children, 71 were eligible to participate and were randomized into the atropine (36) and placebo (35) groups (Fig. 2). Ten subjects (2 atropine and 8 placebo) withdrew after the allocation process. All 61 subjects who continued to participate had at least 2 post-baseline visits, and all their results were used for analyses. The atropine and the placebo groups had similar baseline characteristics in terms of age, SER, and AL, which are shown in Table 1. In addition, the MOFO mfERG responses were also similar in both atropine and placebo groups (Table 2), for all retinal regions and response components, and were independent of the baseline age, SER, and AL (p > 0.05).

**Table 1:** Baseline demographics.

	Atropine (N = 34)	Placebo (N = 27)	
**Gender**			χ2 test (p)
Boys	17	14	0.02 (0.89)
Girls	17	13	
			Unpaired t test (p)
Age (years)	8.6 ± 1.0	8.4 ± 0.8	−0.92 (0.36)
SER (D)	−1.88 ± 1.08	−1.74 ± 0.71	0.60 (0.55)
AL (mm)	24.17 ± 0.79	24.09 ± 0.74	−0.41 (0.69)

**Table 2:** Baseline MOFO mfERG at different retinal eccentricities (mean ± SD).

	Placebo		Atropine	
	Amplitude (nV/deg^2^)	Peak time (ms)	Amplitude (nV/deg^2^)	Peak time (ms)
**Direct component**				
Ring 1 (1.5°)	44.07 ± 19.42	27.21 ± 2.38	47.06 ± 19.01	27.46 ± 2.25
Ring 2 (4.8°)	22.81 ± 9.64	26.70 ± 2.39	22.09 ± 7.76	27.01 ± 1.76
Ring 3 (9.3°)	12.94 ± 5.19	25.96 ± 1.23	14.38 ± 4.74	26.58 ± 1.44
Ring 4 (14.5°)	9.83 ± 4.20	26.17 ± 1.82	11.28 ± 4.44	26.48 ± 1.93
Ring 5 (19.8°)	7.33 ± 3.02	25.97 ± 2.19	8.36 ± 3.51	26.79 ± 2.18
**Induced component**				
Ring 1 (1.5°)	41.65 ± 24.40	39.06 ± 1.66	50.01 ± 27.75	37.95 ± 2.15
Ring 2 (4.8°)	21.88 ± 10.06	38.40 ± 1.36	23.90 ± 12.21	37.30 ± 1.63
Ring 3 (9.3°)	14.23 ± 6.74	37.41 ± 2.11	16.07 ± 9.59	36.97 ± 1.45
Ring 4 (14.5°)	10.52 ± 4.35	36.62 ± 1.53	12.53 ± 8.11	36.25 ± 1.09
Ring 5 (19.8°)	6.61 ± 2.59	36.58 ± 1.72	6.81 ± 4.33	36.09 ± 1.18

No significant difference in any ERG parameters between 2 groups by repeated-measure ANOVA.

DC Amplitude: Ring*Treatment F = 0.38, p = 0.82; Peak time: Ring*Treatment F = 0.89, p = 0.87.

IC Amplitude: Ring*Treatment F = 1.38, p = 0.24; Peak time: Ring*Treatment F = 1.09, p = 0.36.

ERG parameters were independent of baseline age, spherical equivalent refraction, and axial length.

**Figure 3:**
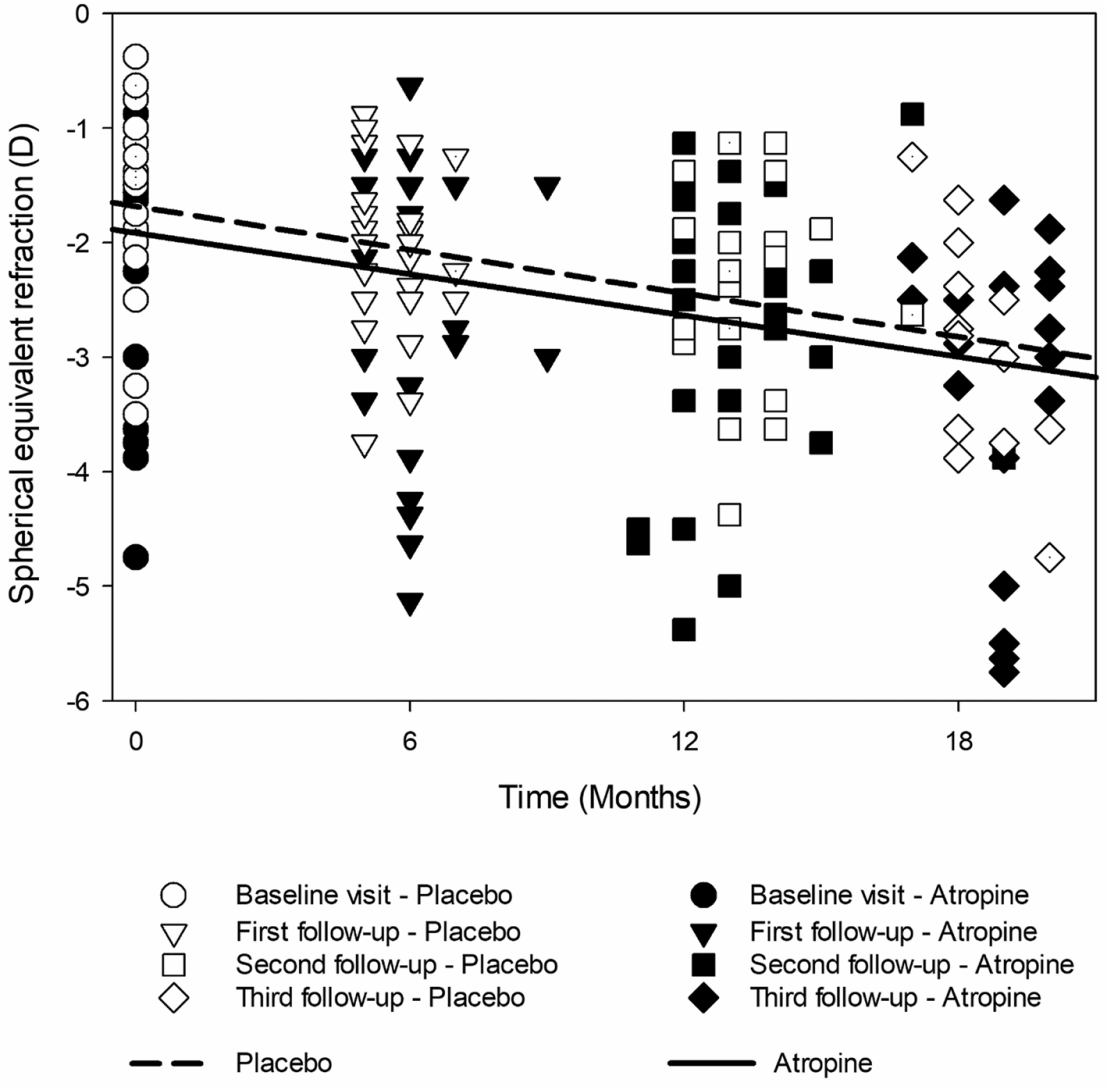
SER over time. Symbols represent individual SER by treatment group and visit, and lines represent mean SER over time by treatment group (as calculated by GLM). The slope of the lines indicates annualized change in SER: placebo group −0.66 (0.41) D, atropine group −0.70 (0.39) D.

The annualized change in SER (± SD) was −0.66 ± 0.41 D/year and −0.70 ± 0.39 D/year, and that in AL was 0.30 ± 0.22 mm/year and 0.32 ± 0.16 mm/year for the placebo and the atropine groups, respectively (Fig. 3). The differences in annualized change for SER and AL between atropine and placebo groups were statistically insignificant (univariate GLM—SER: F = 0.24, p = 0.63; AL: F = 0.43, p = 0.52), after controlling for baseline SER and age.

**Figure 4:**
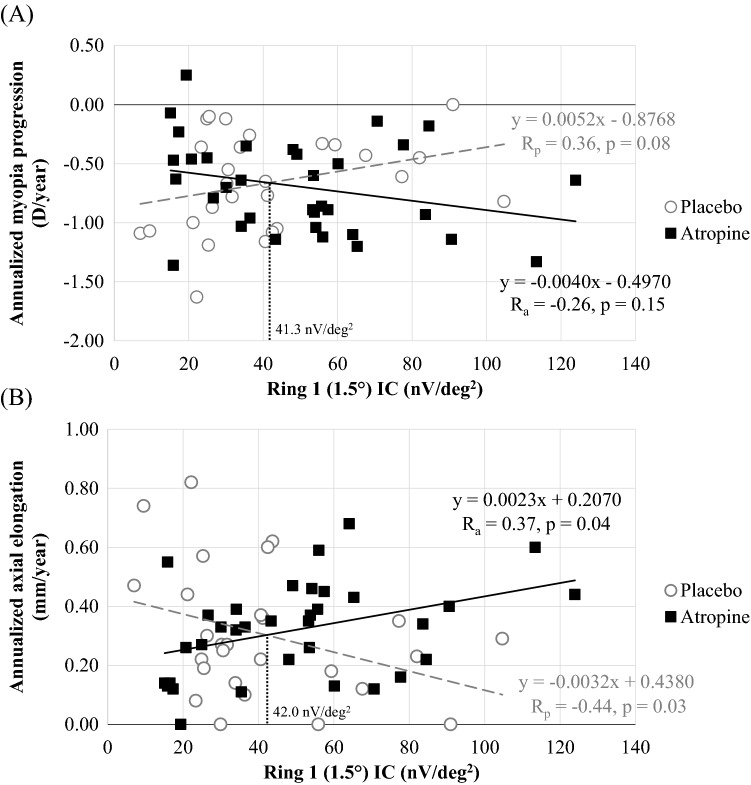
Annualized change in SER and AL by baseline MOFO mfERG response. (**A**) Spherical equivalent refraction; (**B**) Axial length. P-values by Hochberg’s adjustment are presented.

The GLM moderator analysis on treatment group and Ring 1 IC exhibited a significant interaction between treatment and IC for both the annualized change in SER (p = 0.03) and AL (p < 0.01), indicating the regression coefficients for IC on myopia progression in atropine group were significantly different from placebo group. As shown in Fig. 4, the annualized change in AL was negatively correlated with the Ring 1 IC at baseline for subjects in the placebo group (R_p_ = −0.44, p = 0.03). In contrast, annualized change in AL in the atropine group was positively correlated with Ring 1 IC (R_a_ = 0.37, p = 0.04). For the annualized change in SER, although it did not reach statistical significance, similar opposing trends were observed. The correlation coefficients between the placebo and atropine groups were significantly different for SER and AL (R_p_ vs. R_a_—SER: Z = 2.37, p = 0.01; AL: Z = −3.12, p = 0.001). Relationship between other MOFO mfERG parameters and the annualized change in SER and AL are shown in Table 3.

**Table 3:** Relationship between MOFO mfERG and annualized change in SER and AL in the Atropine versus Placebo groups.

	Annualized change in SER			Annualized change in AL		
	Partial correlation (Controlled for baseline SER & age)		Fisher’s test R-to-Z transformation	Partial correlation (Controlled for baseline SER & age)		Fisher’s test R-to-Z transformation
	Placebo [R_p_(p)] (N = 27)	Atropine [R_a_(p)] (N = 34)	R_p_ vs. R_a_ [Z(p)]	Placebo [R_p_(p)] (N = 27)	Atropine [R_a_(p)] (N = 34)	R_p_ vs. R_a_ [Z(p)]
**Direct component**						
Ring 1 (1.5°)	0.25 (0.22)	−0.27 (0.13)	1.96 (0.03)*	−0.40 (0.05)*	0.19 (0.30)	−2.27 (0.01)*
Ring 2 (4.8°)	0.20 (0.34)	−0.20 (0.27)	1.49 (0.07)	−0.14 (0.51)	0.23 (0.21)	−1.38 (0.08)
Ring 3 (9.3°)	0.37 (0.07)	−0.35 (0.05)*	2.77 (0.003)*	−0.30 (0.14)	0.43 (0.01)*	−2.83 (0.002)*
Ring 4 (14.5°)	0.03 (0.89)	−0.33 (0.07)	1.37 (0.09)	0.04 (0.84)	**0.38 (0.03)**	−1.32 (0.09)
Ring 5 (19.8°)	−0.07 (0.74)	−0.23 (0.21)	0.60 (0.27)	0.14 (0.51)	0.34 (0.06)	−0.78 (0.22)
**Induced component**						
Ring 1 (1.5°)	0.36 (0.08)	−0.26 (0.15)	2.37 (0.01)*	−0.44 (0.03)*	0.37 (0.04)*	−3.12 (0.001)*
Ring 2 (4.8°)	0.18 (0.39)	−0.33 (0.07)	1.93 (0.03)*	−0.15 (0.49)	0.37 (0.04)	−1.98(0.02)*
Ring 3 (9.3°)	0.32 (0.12)	−0.30 (0.10)	**2.36 (0.01)**	−0.34 (0.10)	0.34 (0.05)	−2.61 (0.01)*
Ring 4 (14.5°)	0.14 (0.50)	−0.26 (0.15)	1.50 (0.07)	−0.10 (0.62)	0.36 (0.04)	−1.76 (0.04)*
Ring 5 (19.8°)	0.05 (0.83)	−0.28 (0.12)	1.24 (0.11)	0.02 (0.93)	0.42 (0.02)*	−1.57 (0.06)

R_p_: correlation coefficient for the placebo group.

R_a_: correlation coefficient for the atropine group.

*Denotes statistical significance after Hochberg’s adjustment.

To classify a fast progressor (≥ 1.0 D/year), the area under the ROC curve was 0.76 in the placebo group using baseline Ring 1 IC, generating an 89.5% sensitivity and 62.5% specificity. On the other hand, the area under the curve for atropine group was 0.35, or in other words, 0.65 for classifying a slow-progressor, generating a 66.7% sensitivity and 68.0% specificity.

## Discussion

Despite being commonly used for juvenile myopia control, 0.01% topical atropine was not more efficacious than placebo in the current study. However, a significant interaction was noticed between the 0.01% atropine treatment and baseline retinal electrophysiological response. In the placebo group, the baseline Ring 1 IC was negatively correlated with axial elongation (Fig. 4), which was consistent with our previous finding. However, in the atropine group, the relationship between Ring 1 IC and axial elongation reversed, and 0.01% atropine treatment demonstrated a negative efficacy in controlling axial elongation in children with strong baseline MOFO mfERG responses (Table 4).

**Table 4:** Previous prospective, randomized, cohort studies evaluating efficacy of 0.01% atropine in myopia control (Mean ± Standard deviation).

Study	N (atropine)	Age	Baseline SER (D)	SER efficacy (%)	Baseline AL (mm)	AL efficacy (%)
Chia et al., 2012^a^	84	9.5 ± 1.5	−4.47 ± 1.50	43	25.17 ± 0.98	−34
Yam et al., 2019	110	8.2 ± 1.8	−3.77 ± 1.85	27	24.70 ± 0.99	12
Fu et al., 2020^b^	142	9.3 ± 1.9	−2.70 ± 1.64	35	24.58 ± 0.74	29
Current study (all subjects)	34	8.6 ± 1.0	−1.88 ± 1.08	−6	24.17 ± 0.79	−7
Current study _(strong sub-population)_	20	8.6 ± 1.0	−1.90 ± 1.07	−39	24.22 ± 0.95	−40
Current study (weak sub-population)	14	8.6 ± 1.1	−1.85 ± 1.08	20	24.14 ± 0.69	23

$$Efficacy=(Treatment- Control) /Control$$.

^a^Historical placebo control, ^b^no placebo control.

Strong sub-population includes subjects with Ring 1 IC over 41.3 nV/deg^2^ for SER and 42.0 nV/deg^2^ for AL.

Weak sub-population includes subjects with Ring 1 IC below 41.3 nV/deg^2^ for SER and 42.0 nV/deg^2^ for AL.

The lack of efficacy in reducing myopia progression may be due to the young age and low myopic refractive error in the current study population. Table 4 also compares the results of the current study with three previous randomized controlled trials testing 0.01% atropine. Across the four studies, 0.01% atropine efficacy appeared to be better in older children with more myopia at baseline. Consistent with the findings of this study, it was previously reported that atropine was less effective in children of younger age in both high^22^ and low concentrations^23^. Our results further confirmed earlier findings that the baseline central IC in MOFO mfERG^18^ was associated with future myopia progression in young children with low myopia in the absence of atropine treatment. While the area under the curve was 0.76 for baseline Ring 1 IC, but 0.58 and 0.44 for baseline SER and AL, respectively, for classifying a fast progressor, MOFO mfERG may offer better additional predictive value than only baseline SER or AL. Interestingly, 0.01% atropine treatment reversed the association between Ring 1 IC and axial elongation from a negative correlation to a positive one (Table 3). To illustrate this phenomenon, the atropine group was further divided into strong and weak Ring 1 IC sub-groups based on the intersection points as shown in Fig. 4. In the weak sub-group, the 0.01% atropine showed a positive efficacy in controlling myopia. However, in the strong sub-group, the 0.01% atropine showed a negative efficacy when compared with placebo (Table 4). As our results indicated that 0.01% atropine could have opposite effect on children with different MOFO mfERG response at baseline, it is suggested that population reference ranges should be established for retinal responses to facilitate the classification of an appropriate individualized myopia control strategy. We also speculate that prior screening may be needed for other myopia control methods, such as higher concentrations of atropine treatment^13^ and manipulation of peripheral optical defocus^5, 6, 27^.

Atropine, a non-selective anti-muscarinic agent, is hypothesized to target retinal neurons for its myopia control mechanism. However, the reported effects of atropine on ERG have been inconsistent, potentially due to cross-study differences in atropine concentration, administration frequency, treatment duration, and subject age. One drop of 0.1% atropine was reported to decrease the dark-adapted oscillatory potential and delay the peak time of full-field ERG^28^. In contrast, 24-month daily treatment with 0.01% atropine in myopic children, and a 2-week course of 0.01% atropine in emmetropic adults had minimal effect on full-field and pattern ERG, respectively. Moreover, the mfERG second-order kernel (mainly reflecting inner retinal function) amplitude was similar in myopic children receiving 24-month daily 1.0% atropine when compared with the placebo group^29^. Although not being investigated in the current study, the effect of chronic atropine treatment on MOFO mfERG, as well as interaction on refractive error development is warranted.

Cycloplegia and mydriasis have been the most frequently reported adverse effects of atropine^30^, causing difficulty in near-distance work and photophobia. Other rarer complications include allergy, headache, chalazion, and systemic side effects. In the current study, no forementioned adverse effect was reported, consistent with the favorable tolerability of 0.01% atropine in other studies^8–10^. However, due to the significant interaction effect between atropine treatment and baseline mfERG on axial elongation, as well as myopia progression, 0.01% atropine ought to be used with caution on subjects with strong baseline retinal response, whose progression may be worsened over natural history according to our results. In terms of clinical application, therefore, patient selection is critical, and care should be exercised when prescribing 0.01% atropine for myopia control, particularly when considering young children with low myopic refractive error. In addition, a potential reason for difference in central IC amplitudes between subjects at baseline may be related to the M to L cone ratios which was suggested to be associated with myopia development in chick eyes^31, 32^. Further investigations in different aspects, e.g., possible mechanisms, are necessary.

This study has several limitations. The sample size was relatively small in the current study when compared with other studies. With the small sample, the effect size and statistical power for the interaction between MOFO mfERG and treatment groups, i.e., the moderator terms including age and baseline refraction in the GLM models, were 0.15 and 87%, respectively, for annualized change in AL, but only 0.09 and 62%, respectively, for annualized change in SER. The follow-up visits of the subjects did not take place within the planned time-windows due to a lockdown caused by the COVID-19 pandemic. Instead of a direct comparison of SER and AL by visit, annualized change was compared between atropine and placebo groups in the current study to account for delays in visit schedules incurred by the pandemic-related lockdown. The evaluation of myopia control efficacy could also be affected by the lockdown effect in the current cohort due to the COVID-19 pandemic^24–26^. The increased time spent indoor and reduced time spent outdoor might have diluted the difference between the atropine and placebo groups. Monitoring before the commencement of treatment can also be implemented to ensure the fast vs. slow progressors ratio was similar between groups.

To conclude, although low-concentration (0.01%) atropine is being used as a common pharmacological strategy in myopia control, its efficacy in the current study differed from results of previous studies. Moreover, 0.01% atropine efficacy was found to vary based on retinal function at baseline. Careful selection of candidates for 0.01% atropine treatment is important, together with consideration of other myopia control strategies. The assessment of central inner retinal function may be essential as an objective indicator when selecting the appropriate treatment strategy for juvenile myopia control. Further studies are also warranted to investigate whether this interaction was specific to the 0.01% atropine, or would extend to other regimes of atropine (e.g., higher dosage of 0.05%).

## Data Availability

The data supporting the results reported in the article are available at https://doi.org/10.17605/OSF.IO/5BYRH.
